# Identification of agro-physiological traits of lentil that reduce risks of drought

**DOI:** 10.3389/fpls.2022.1019491

**Published:** 2022-10-24

**Authors:** Abeya Temesgen Tefera, Garry J. O’Leary, Thabo Thayalakumaran, Shiwangni Rao, Viridiana Silva-Perez, Arun S. K. Shunmugam, Roger Armstrong, Garry M. Rosewarne

**Affiliations:** ^1^ Agriculture Victoria Research, Grain Innovation Park, Horsham, VIC, Australia; ^2^ Centre for Agricultural Innovation, The University of Melbourne, Parkville, VIC, Australia; ^3^ Agriculture Victoria Research, Centre for Agri Bioscience, Melbourne, VIC, Australia; ^4^ Department of Animal, Plant and Soil Sciences, La Trobe University, Melbourne, VIC, Australia

**Keywords:** APSIM, drought, ideotype breeding, lentil, phenological traits, physiological traits

## Abstract

Ideotype breeding is an essential approach for selection of desired combination of plant traits for testing in crop growth model for potential yield gain in specific environments and management practices. Here we parameterized plant traits for untested lentil cultivars for the APSIM-lentil model in phenology, biomass, and seed yield. We then tested these against independent data and applied the model in an extrapolated analysis (i) to assess the impact of drought on productivity across different rainfall environments; (ii) to identify impactful plant traits and (iii) to design new lentil ideotypes with a combination of desirable traits that mitigate the impact of drought, in the context of various agronomic practices across a wide range of production environments. Desirable phenological and physiological traits related to yield were identified with RUE having the greatest effect on yield followed by HI rate. Leaf size significantly affected seed yield (p< 0.05) more than phenological phases. The physiological traits were integrated into four ideotype designs applied to two baseline cultivars (PBA Hallmark XT and PBA Jumbo2) providing eight ideotypes. We identified a combination of genetic traits that promises a yield advantage of around 10% against our current cultivars PBA Hallmark XT and PBA Jumbo2. Under drought conditions, our ideotypes achieved 5 to 25% yield advantages without stubble and 20 to 40% yield advantages with stubble residues. This shows the importance of genetic screening under realistic production conditions (e.g., stubble retention in particular environments). Such screening is aided by the employment of biophysical models that incorporate both genetic and agronomic variables that focus on successful traits in combination, to reduce the impact of drought in the development of new cultivars for various environments. Stubble retention was found to be a major agronomic contributor to high yield in water-limiting environments and this contribution declined with increasing growing season rainfall. In mid- and high-rainfall environments, the key drivers of yield were time of sowing, physiological traits and soil type. Overall, the agronomic practices, namely, early sowing, residue retention and narrow row spacing deceased the impact of drought when combined with improved physiological traits of the ideotypes based on long term climate data.

## Introduction

Lentil is a cash crop and provides rotational benefits in cereal-based farming system (Moodie and Brand, 2019). The crop is predominately adapted to the semi-arid regions of temperate Australia as well as globally (Thomson et al., 1997; Siddique et al., 1998; Delahunty et al., 2018; Sadras et al., 2021; ). In low (<300mm) and medium (300-450mm) rainfall zones of South-eastern Australian environments, lentil production heavily relies on seasonal conditions (amount and timing of rainfall and extreme temperatures). The region is generally characterized as a semi-arid environment (Clarke et al., 2021) with a wet early crop growth stage and a dry finish (Rodriguez and Sadras, 2007; Sadras and Rodriguez, 2007; Flohr et al., 2017; Hunt et al., 2019; Flohr et al., 2021). However, the early growing season rainfall is unpredictable and often delays sowing. Any soil water stored during early crop growth is usually depleted due to high rates of soil evaporation and increased vapor pressure deficit during subsequent crop reproductive growth in spring (Rodriguez and Sadras, 2007; Sadras and Rodriguez, 2007). As a result, water stress and drought during the reproductive phase has considerable effect on seed yield.

Previous studies have shown the advantage of agronomic practices, such as stubble retention in improving soil water storage by reducing losses through evaporation in the dry environments. For example, Page et al. (2019) reported 44 mm of more soil water in stubble retention treatment than a plot without stubble in wheat. Improved water infiltration in a stubble retention plot over the removed stubble was also seen using blue dye techniques under field condition (Roper et al., 2013). In another study, stubble retention plot had 59 mm less soil evaporation than bare soil plot at 250 mm rainfall (Monzon et al., 2006).

However, in environments, where multiple constraints are limiting crop production (Sadras et al., 2002; Nuttall et al., 2003; Rodriguez et al., 2006; Armstrong et al., 2009; O'Leary et al., 2016), a key understanding of agronomic and physiological processes can aid selection and optimization of adaptive plant traits for drought stress (Chenu et al., 2013; Kaloki et al., 2019) and other related future climatic conditions (Hammer et al., 2006; Hammer et al., 2014; Semenov et al., 2014).

Several studies have used crop growth models to understand the interactions between plant trait by environment by agronomic practice for desirable trait optimization under water limited environments (Chenu et al., 2013; Casadebaig et al., 2016; Chenu et al., 2017; Solomon et al., 2017; Ibrahim et al., 2019; Xin and Tao, 2019; Liu et al., 2020). This approach to assist ideotype breeding offers good prospects for evaluating plant desirable traits for specific adaptation. The concept of ideotype breeding - an ideal model in cultivar development - was first introduced by Donald (1968) and has been applied for selection of multiple plant traits in combinations for testing in crop growth models for potential yield gain in specific production circumstances.

Using global sensitivity analysis and crop simulation modelling techniques, Casadebaig et al. (2016) have assessed the impact of multiple wheat traits on yield and yield related parameters across different environments. Overall, their study classified multiple traits into no impact, low impact and high impact traits. They found several traits, including phenology, leaf area, radiation use efficiency, harvest index and root related parameters, that impacted positively on crop yield most of the time. These traits together with the existing agronomic practices have been proposed to maximize yield and stability of commercial varieties across various environments. However, adding traits to form an ideotype is not straight forward because of non-linearity and interaction among traits for a particular environment, including management options (Archontoulis et al., 2014; Casadebaig et al., 2016; Gouache et al., 2017; Chenu et al., 2018; Kaloki et al., 2019; Xiao et al., 2020; Collins and Chenu, 2021; Ojeda et al, 2021; Ojeda et al, 2022).

In this study we developed new parameters for untested lentil cultivars for the APSIM-lentil model in phenology, biomass, and seed yield. We then tested these against independent data and applied the model in an extrapolated analysis (i) to assess the impact of drought on productivity across different rainfall environments; (ii) to identify impactful plant traits and (iii) designed eight new lentil ideotypes with a combination of desirable traits that mitigate the impact of drought, in the context of various agronomic practices across a wide range of production environments.

## Materials and methods

### Field experiments

Three locations selected for field experiments were Hopetoun (35°42’10”S 142°27’26.0”E) and Beulah (35°59’52.5”S 142°33’12.7”E), representing low rainfall (< 300mm annual) environments, and Horsham (36°44’10.8”S 142°06’25.4”E) which represented a medium rainfall environment (300 – 400 mm) in Victoria, Australia. In 2020, time of sowing trials were carried out at Horsham and Hopetoun for model parameterization and testing (Experiment 1). Field experiments conducted between 2016 and 2020 at Horsham and Beulah were used for model validation (Experiment 2).

#### Experiment 1

Five commercially released varieties (PBA Jumbo2, PBA Kelpie XT, PBA Hallmark XT, PBA Ace & Nipper), and one breeding line (12H681L-5-15HSHI3012) of lentil were evaluated at three times of sowing (TOS) where TOS1 was on the 28^th^ of April, TOS2 on the 18^th^ May and TOS3 on the 28^th^ of May, representing early, optimum and late time of sowings) at each location in 2020. The experiments were arranged in a split-plot design with three replications with time of sowing as main-plots and genotype as sub-plots.

#### Experiment 2

Independent field trials were carried out at Horsham and Beulah between 2016 and 2020. The trials were conducted as Randomized Completed Block Designs, with three replicates. The datasets of five varieties (matching experiment 1) were used for model validation for days to 50% flowering and seed yield.

The trials were planted at 120 plants per m^2^ in both Experiment 1 and Experiment 2. The experimental unit area was 5m x 1.25m with five rows per plot. Crop management practices, disease, weed and insect controls were carried out as per farmer best practices.

The datasets from these two experiments were used for parameterizing (Experiment 1) and testing (Experiment 2) the ability of the APSIM-lentil model to predict seed yield and yield related parameters of new varieties of lentil.

### Model inputs

There are several modules linked to the APSIM model to simulate crop growth and development. Cultivar, climate and soil modules contain variety and site-specific parameters connected to the central system of the model. The key specific model parameters, including (i) soil information (soil type, soil hydraulic properties, initial soil water & nutrient status), (ii) crop and genotype specific coefficients and (iii) daily climate information (Tmax, Tmin, rainfall, solar radiation) are required to simulate crop growth and development. The key physiological principles applied to all crops for simulation are the same across different crop species except some specific parameters, such as the genotypic coefficient, can be measured and included into the cultivar module through parameterization (https://www.apsim.info/documentation/model-documentation/crop-module-documentation/plant/).

### Soil datasets

The soil was sampled at soil depths of 0-15, 15-30, 30-50, 50-70, 70-90, 90-110 and 110-130 cm at Hopetoun and Horsham for soil analysis. At each location, three replications (soil cores) were taken by moving in diagonal line across the experimental paddock and taking three soil cores at equal points. Detailed information and methods of data analysis for the soil parameters followed the analytical laboratory procedure (CSBP Lab Method, 2019).

Soil water content at sowing were determined using the gravimetric method at the following soil depths: 00-15, 15-30, 30-50, 50-70, 70-90, 90-110 and 110-130. The samples were oven dried at 105°C for 48 hours and soil water content was determined following the soil matters book procedure (Dalgliesh and Foale, 1998). The data were used for crop simulation modelling initial set up.

### Climate datasets

Meteorological data (rainfall, radiation, minimum and maximum temperature) was measured daily at each experimental site using an automatic weather station (https://greenbrain.ag/documents/mea-weather-station-brochure.pdf) located within 0.5-km radius of the trial.

### Cultivar datasets

#### Model parameterization

Cultivar specific parameters were measured on six varieties of lentils sown at the optimum time and were subsequently used for model parameterization. Harvest index rate, canopy height, stem development rate, main stem node number, leaf area, and thermal time dependent phenology were measured on five plants of each variety.

##### Node appearance rate and phenology

The number of nodes were counted weekly from Experiment 1 and the data collected were used to determine the node appearance rate and growth stages as described in the APSIM cultivar module (x_node_no_app vs y_node_app_rate).

Key phenological stages were recorded and the duration between each stage was determined by cumulative thermal time (growth degree days) except the period between sowing to germination, which is governed by soil moisture and soil temperature (Keating et al., 2003; Archontoulis et al., 2014). Sowing to emergence growth is driven by seed depth and thermal time, in addition to water content of the soil (Robertson et al., 2002). The cumulative thermal time during this phase is estimated from seeding depth, shoot rate and the time lag during shoot elongation.

Cumulative thermal time in growth degree days (GDD) between each growth stage was calculated by summing daily values of thermal time, taking into account the three cardinal temperatures: 0.0 (base temperature or the lower limit), 30.0 (optimum for growth), and 40.0°C (the upper limit) using a sine curve approach (Jones and Kiniry, 1986), where the model accounted growth in lentil when the temperatures for thermal time were between 0°C and 40°C.

Phenologically, the end of juvenile stage was measured when the 8^th^ node appeared, and floral initiation at the 14^th^ node. Flowering and physiological maturity stages of the crop were recorded when 50% of the plants in the plot had at least one flower open and when 90% of the plants and pods had turned yellow. Aboveground biomass was also harvested six times between the start of grain filling and physiological maturity to determine harvest index rate.

##### Cultivar specific physiological parameters

We parameterized cultivar specific parameters related to biomass accumulation and seed yield from field trials conducted under optimum field conditions. These parameters were radiation use efficiency (RUE), harvest index rate and leaf size.

###### Harvest index rate

During the seed formation phase, aboveground biomass was harvested between start of grain filling and physiological maturity to determine harvest index (the ratio of seed yield to total aboveground dry matter). The daily harvest index rate was estimated from the slope of the line drawn between days after sowing and harvest index.

###### Leaf area

Leaf area was measured on 90 representative leaf samples of each variety using a leaf area meter (LI-3100C, USA) along with the number of nodes at different growth stages.

###### Intercepted photosynthetically active radiation and radiation use efficiency

Incident and upcoming photosynthetically active radiation (400-700nm) were measured using a ceptometer (AccuPAR LP-80; Decagon Devices, Inc., USA) once every fortnight on the TOS2 trial. The fraction of intercepted photosynthetically active radiation (fiPAR) was calculated as in eq.1. The daily cumulative iPAR was estimated from daily solar radiation multiplied by daily fiPAR. The RUE was estimated from the slope of the line drawn between aboveground biomass and cumulative intercepted photosynthetic active radiation for each genotype to determine daily biomass accumulation in the model (Verón et al., 2005).


$$\text{iPAR}=1-(\frac{\text{transmitted }}{\text{incident }})\text{ (eq}.1)$$

### Management practices and model initial conditions

The model was used to simulate various parameters of selected lentil varieties to time of sowing experiments under different environmental conditions at various TOS’s in Hopetoun and Horsham in the 2020 and 2021 growing seasons. Other datasets derived from National Lentil Breeding Program trials conducted at Beulah and Horsham between 2016 and 2020 were also used for validation. The same agronomic practices from field experiments conducted at Hopetoun and Horsham in 2020 and 2021 crop growing seasons were used in relation to sowing characteristics, plant population, type and rate of fertilizer. Further information of the APSIM lentil module is found at: https://www.apsim.info/documentation/model-documentation/.

### Measures of model performance

The APSIM model capability of reproducing the experimental data in lentil trials was tested using the root mean square error (RMSE), normalized root mean square error (NRMSE), Pearson coefficient (R), Nash Sutchiffe model efficiency coefficient (ME), index of agreement (d) and Lin’s concordance correlation coefficient (CCC) by comparing the measured and modelled values. The model accuracy was evaluated as indicated in **Table 1**.

**Table 1:** Indicator of model.

Error terms	Model accuracy	Reference
Pearson coefficient (R)	Closest to unity (more accurate)	Bosi et al. (2020); Rahimi-Moghaddam et al., 2021
Index of agreement (d)	unsatisfactory (d ≤ 0.75), satisfactory (0.75< d ≤ 0.85), good (0.85< d ≤ 0.95) and very good (d > 0.95)	Willmott (1981); Bosi et al. (2020)
Normalized root mean square error (NRMSE)	NRMSE=0 (higher accuracy of the model)	Rahimi-Moghaddam et al., 2021
Root mean square error (RMSE)	RMSE ~ 5 (good for days to 50% flowering); RMSE ~ 0.5t/ha (good for seed yield); RMSE=1.5t/ha (good for biomass)	Kirkegaard et al. (2016); Robertson and Lilley (2016)
Nash Sutchiffe model efficiency coefficient (ME)	unsatisfactory (NSE ≤ 0.5), satisfactory (0.5< NSE ≤ 0.65), good (0.65< NSE ≤ 0.75) and very good (NSE > 0.75)	Moriasi et al. (2007)
Lin’s concordance correlation coefficient (CCC)	ideal = 1, lower values indicate bias from the y = x line	Lake et al. (2021)

Adapted from Gomes et al. (2020) with modification.

### Scenario analysis

**Figure 1:**
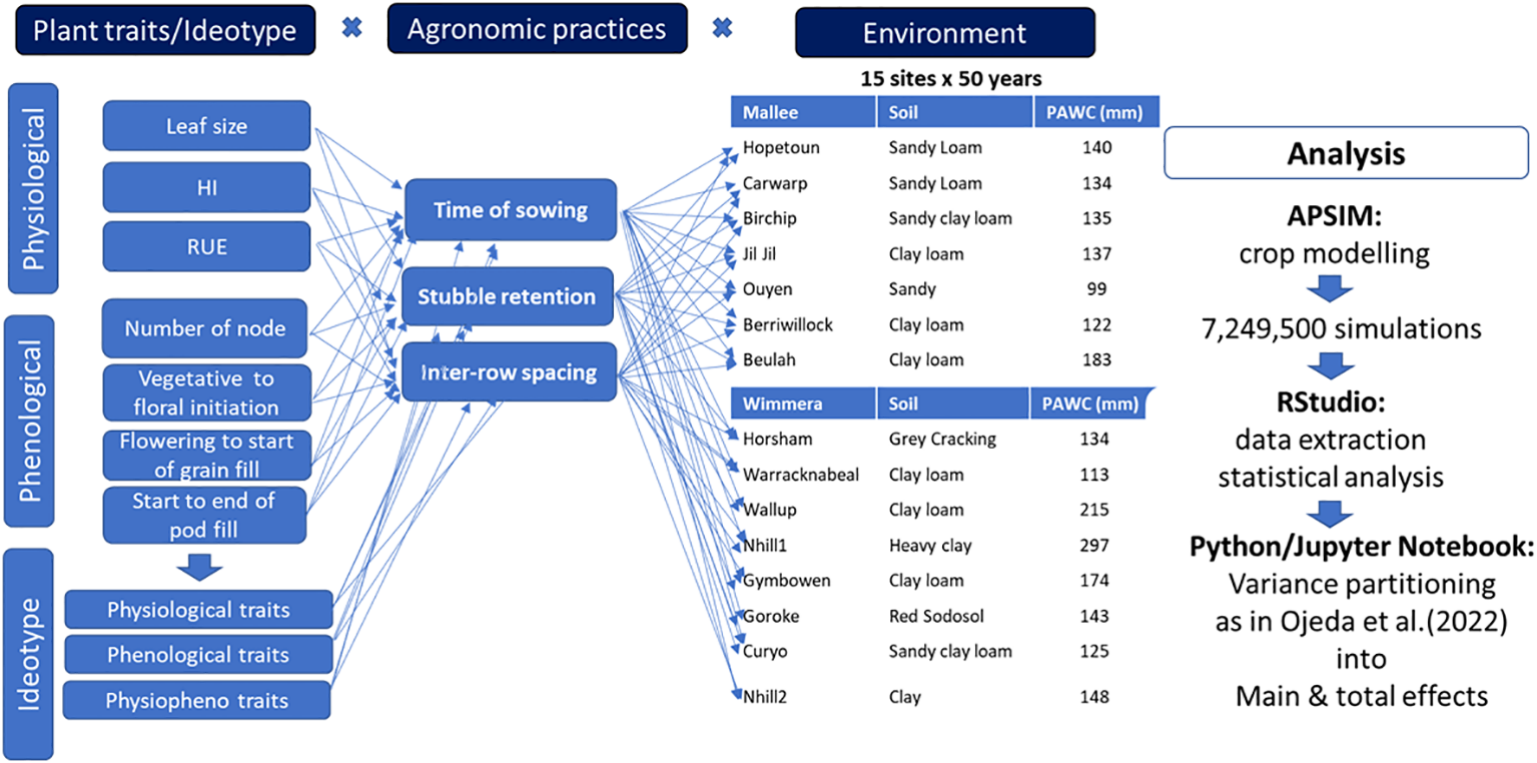
Framework of ideotype x argonomic practices x environmental analysis for plant traits optimization in lentil.

A factorial combination of plant traits/ideotype and agronomic practices (time of sowing, stubble retention, and row spacing) was designed and run using the APSIM-lentil model across multiple environments. Five times of sowing (TOS1=April 28, TOS2=May 12, TOS3=May 26, TOS4=June 7, TOS5=June 23), two stubble management (0 and 6 t/ha of initial surface residue of wheat), two row spacings (narrow row spacing = 19cm & wide row spacing = 38cm) and two varieties of lentil (Jumbo2 and Hallmark XT), including ideotypes and physiological traits were included in the scenario analysis. Overall, 7,249,500 simulated seed yields of lentil were generated using 50-years of historical climate data across 15-sites in Wimmera and Mallee environments in Victoria. **Figure 1** presents the workflow framework of the scenario analysis.

Based on trait-yield relationship, potentially important plant traits were identified, and genetic diversity of the traits were created by a ten percent increase (10%-40%) around the baseline variety. The traits were combined with best-bet agronomic practices and evaluated across multiple sites and years for yield and plant available water improvement. In this study, 750 environments were clustered into three rainfall conditions (namely., low, medium and high) using percentiles. The average yield of high rainfall environments were used to compare to average water-limited yield potential of each region and quantify the impact of drought. Collins and Chenu (2021) also used average water-limited yield potential and average yield from no water stress environment to estimate drought-induced yield loss (%) for each region. The output generated from the APSIM model was then extracted using RStudio and the source of variance of simulated yield driven by ideotype, agronomic practices and environments were partitioned into main and total effects. The degrees at which each factor contributed to simulated yield and plant available water variance were estimated using the main effect. The interaction effect was explained from total effects.

The formula of these sensitivity indices are presented in eq 2 and eq 3 as in (Ojeda et al. 2021; 2022) and Webber et al. (2018).


$${\text{ME}}_{i=}\frac{Variance(E[Yield\;X_{i}])}{Variance\;(Y)}$$

$${\text{TE}}_{i\;=1\;-}(\frac{Variance(E[Yield\;X_{-i}])}{Variance\;(Y)}$$

Where, E[YieldXi] = yield across all source of variance X_i_; E[YieldX-i] = yield across all sources of variance without X_i_; Contribution of each factor to yield variability is explained by the main effect (ME), while the interaction effect is explained by total effect (TE), a factor with a high proportion of the total variance is highly interactive with other factors.

## Results

### The lentil model

A new APSIM-lentil model using an existing APSIM-chickpea background (Robertson et al., 2002) has been recently introduced into the latest classical version of APSIM 7.10. However, initial testing showed that new lentil cultivars were not simulated well, and cultivar specific parameters were needed to be empirically determined. Briefly, the lentil model follows the family of pulse models in APSIM that simulate phenological development, growth and seed yield on a daily time step basis (Keating et al., 2003; Holzworth et al., 2014; Robertson and Lilley 2016). Together with environmental factors of weather conditions (rainfall, solar radiation, temperature and vapor pressure) the model simulates a soil water and nitrogen balance that are important factors involving crop response to drought.

### Model parameterization

#### Soil specific parameters

Soil parameters used to simulate Hopetoun and Horsham experiments were collected and converted into APSIM format (**Supplementary Table 1**). Drained upper limit (DUL) and crop lower limit (CLL) were assumed to be equal to soil water measured using a pressure plate in a laboratory at -0.33bar (field capacity) and at -15bar (LL15, permanent wilting point) matric pressure, respectively. Saturated soil water content was estimated from soil bulk density and total soil porosity, as described by Dagliesh and Foale (1998). The measured soil C:N ratios at Hopetoun and at Horsham were added to APSIM for simulation.

In addition to the soil parameters presented in **Supplementary Table 1**, copies of soil descriptions similar to the soil textures of Hopetoun and Horsham in APSIM were chosen and the default values were used to represent the drainage coefficient, root water coefficient, root penetration parameter and soil water conductivity. The Beulah site soil specific datasets were used from APSOIL data base (APSIM version 7.10). In addition, a total of 14 previously characterized soils of Wimmera and Mallee were used for scenario analysis.

#### Cultivar specific parameters

Cultivar specific key physiological and phenological parameters of lentil were parameterized using field measured datasets. The number of nodes formed on the mainstem varied between 25 and 28 at 1450 °Cd (148 days after emergence) in six lentil genotypes evaluated in experiment 1. Node appearance rates of high yielding genotypes (PBA Jumbo2 and PBA Kelpie XT) were slightly lower compared to the rest of the genotypes at early reproductive stage (i.e., 14^th^ node, floral initiation). Overall, the node appearance on mainstem of lentil was linearly and positively associated with growth degree days (R = 0.97 - 0.98, p< 0.05), where PBA Jumbo2 and 12H681L-5-15HSHI3012 had relatively slower node appearance rates (**Supplementary Table 2**). The stay green late maturing genotypes (Nipper, PBA Ace, PBA Hallmark XT) tended to have faster node appearance rates.

Days to 50% emergence ranged from 15 (TOS1) to 19 days (TOS3) with the corresponding accumulated daily degree days of 171 (TOS1) – 179 °Cd (TOS3) at 50mm sowing depth (Experiment 1). However, the model underestimated days to emergence using the default values. For example, 8 days for TOS1 and 9 days for TOS3 were simulated using the default values of pre-emergence shoot rate (1 °Cd/mm) and shoot lag (15 °Cd) at a sowing depth of 50 mm.

The accuracy of model prediction for days to emergence was improved (**Supplementary Figure 1**) by parameterizing with phenotypic values (**Supplementary Table 2**) for developing shoot rate, shoot lag and thermal time from nine field trials sown at two sowing depths (SD15 = 15mm and SD50 = 50mm).

All the tested varieties reached the end of juvenile stage at the same degree days except for Nipper and PBA Jumbo2. PBA Ace, PBA Hallmark XT and 12H681L-5-15HSHI3012 reached floral initiation at the same time and this was earlier than Nipper, PBA Kelpie XT and PBA Jumbo2. In terms of days from emergence, PBA Ace and PBA Hallmark XT were the first to reach floral initiation compared to PBA Kelpie XT, 12H681L-5-15HSHI3012 and Nipper (**Supplementary Table 2**).

The duration from emergence to the start of pod filing was estimated from a fitting line between harvest index and days after emergence. PBA Kelpie XT, PBA Jumbo2 and 12H681L-5-15HSHI3012 started pod filling earlier than the other genotypes. These genotypes also had a longer pod filling phase compared to PBA Ace, PBA Hallmark XT and Nipper.

The proportion of biomass converted to seed yield is determined by a cultivar specific harvest index rate. This was calculated from the slope of the fitted line between days after sowing and harvest index in each genotype. There was a slightly higher harvest index rate in PBA Kelpie XT and PBA Jumbo2 than the remaining genotypes (**Supplementary Table 2**). PBA Jumbo2 recorded the highest RUE while the 12H681L-5-15HSHI3012 line had the lowest (**Supplementary Table 2**). The remaining genotypes had relatively similar RUE.

The maximum individual leaf area (max leaf size) was also included in lentil parameterization using the TOS2 trial at Horsham 2020. Pooled leaf size (n=90) for each variety was calculated at the vegetative stage (end of juvenile stage, 8^th^ node) and at floral initiation (14^th^ node). PBA Jumbo2, PBA Kelpie XT and PBA Ace had higher individual leaf area than the rest of the genotypes (**Supplementary Table 2**), while Nipper had the smallest leaf size at both growth stages.

The calculated coefficients for each cultivar (shaded rows, **Supplementary Table 2**) along with published data (unshaded area of the **Supplementary Table 2**) were used for APSIM-lentil model parameterization. These cultivar descriptions were added into the APSIM-lentil module.

### Model evaluation

#### Phenology

Datasets of seven field trials conducted in two locations were used for model testing. There was good agreement between measured and simulated data for days to flowering (DF) at Horsham and Beulah and physiological maturity (DM) at Horsham for all 6 varieties. The RMSEs were less than 5 days for DF at each season and the model predicted DF with high accuracy across all seasons at both Horsham (RMSE = 0.54 days, NRMSE= 0.22, ME = 0.34 days, d = 0.88, R = 0.83) and Beulah (RMSE = 2.67days, NRMSE= 0.02, ME = 0.30, d = 0.79, R = 0.67) (**Supplementary Figures 2A, B**). Physiological maturity was also simulated with acceptable accuracy (RMSE = 4.26 days, NRMSE= 0.02, ME = 0.68, d = 0.94, R = 0.93) at Horsham.

#### Biomass

The response of biomass to time of sowing of six varieties of lentils were accurately simulated (**Supplementary Figures 3i, 3ii**) and delayed sowing (TOS3) reduced biomass of each variety. Predictions of this trait were therefore slightly more underestimated in late sowing than in early sowing experiments.

Overall, the APSIM-lentil model predicted biomass yield with high accuracy in both early (RMSE = 0.89 t/ha, NRMSE= 0.40, ME = 0.92, d = 0.98, R = 0.96) and late (RMSE = 0.61 t/ha, NRMSE= 0.25, ME = 0.96, d = 0.98, R = 0.98) time of sowing as shown in **Supplementary Figure 3** (i and ii).

#### Grain yield

The APSIM-lentil model was evaluated for six genotypes using datasets from ten field trials. Measured and simulated grain yields were compared for different genotypes, time of sowings, seasons, locations, and soil types. Measured seed yields across the seasons ranged from 1.4 - 4.19 t/ha at Horsham, 1.66 - 2.29 t/ha at Hopetoun and 0.78 - 3.93 t/ha at Beulah compared to simulated yields from 1.61 - 4.33 t/ha at Horsham, 1.31 - 2.88 t/ha at Hopetoun and 1.77 - 4.20 t/ha at Beulah.

**Figure 2:**
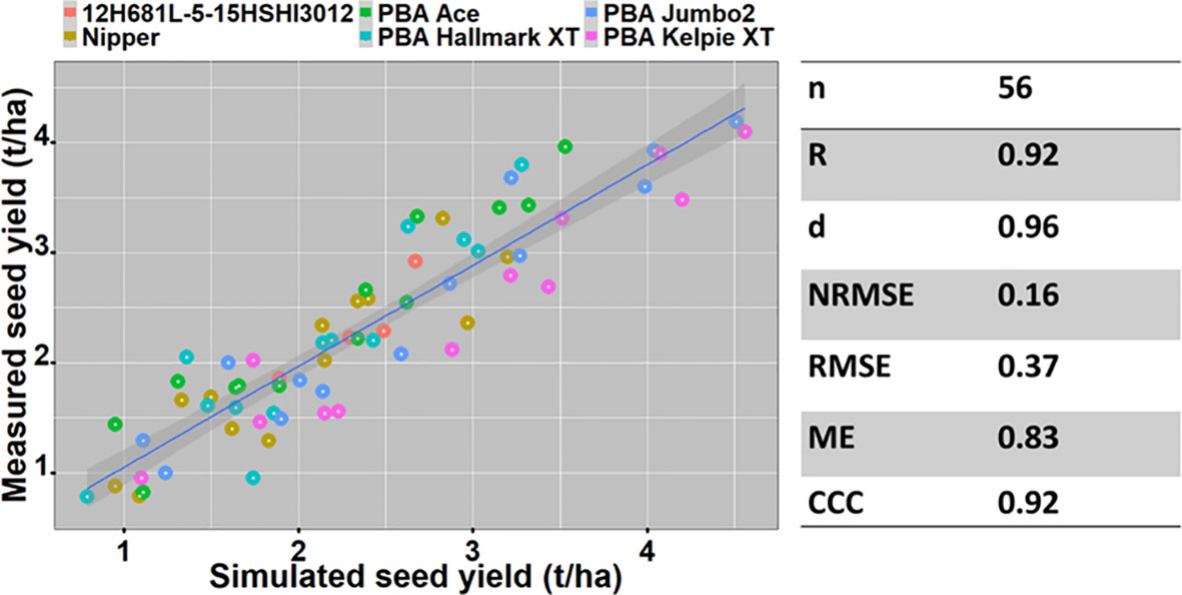
The relationship between measured and simulated yield of six lentil varieties between 2016 & 2020 at Horsham and 2016 & 2020 (except in 2018) at Beulah.

The model predicted seed yield with high accuracy (**Figure 2**) using datasets of six varieties across locations (Horsham, Hopetoun and Beulah) and years (2016 – 2020).

### Screening of plant traits for ideotype designing

Pearson correlation was applied to assess the associations between plant traits and yield. Desirable phenological and physiological traits related to yield were identified (**Table 2**) with RUE having the greatest effect on yield followed by HI rate. Leaf size significantly affected seed yield (p< 0.05) more than phenological phases. The physiological traits were integrated into four ideotype designs applied to two baseline cultivars (PBA Hallmark and PBA Jumbo2) providing eight ideotypes from four increasing increments of 10% over baseline cultivar values. The phenological parameters defining the phenological traits of the baseline cultivars were used for each respective four ideotypes. These were combined with agronomic practices for productivity optimization and risk reductions related to drought.

**Table 2:** The relationship between yield and phenological and physiological traits.

		Productivity	Phenological traits							Physiological traits				
				Seed yield	Emergence to end of juvenile	End of juvenile to floral initiation	Floral initiation to flowering	Flowering to start of pod fill	Start to end of pod fill	Emergence to flowering	Flowering to maturity	Node appearance rate	Harvest index rate	Radiation use efficiency	Leaf size at 8th node	Leaf size at 14th node
Productivity	Seed yield	1.00												
Phenological traits	Emergence to end of juvenile	-0.12	1.00											
	End of juvenile to floral initiation	0.58	-0.83*	1.00										
	Floral initiation to flowering	-0.10	0.52	-0.67	1.00									
	Flowering to start of pod fill	-0.78	-0.51	0.05	-0.29	1.00								
	Start to end of pod fill	0.14	-0.27	0.43	-0.81	0.07	1.00							
	Emergence to flowering	-0.09	-0.36	0.31	-0.68	0.30	0.92	1.00						
	Flowering to maturity	-0.43	0.16	-0.23	-0.41	0.29	0.75	0.81	1.00					
Physiological traits	Node appearance rate	0.21	-0.79	0.59	0.06	0.29	-0.22	-0.10	-0.50	1.00				
	Harvest index rate	0.69	-0.13	0.53	-0.55	-0.52	0.52	0.31	-0.02	-0.24	1.00			
	Radiation use efficiency	0.64	0.55	-0.19	0.34	-0.93**	0.00	-0.12	-0.11	-0.39	0.52	1.00		
	Individual leaf area at 8th node	0.93**	-0.17	0.61	-0.13	-0.69	-0.04	-0.30	-0.64	0.23	0.67	0.48	1.00	
	Individual leaf area at 14th node	0.86*	-0.33	0.61	-0.19	-0.58	0.31	0.23	-0.26	0.26	0.77	0.60	0.74	1.00

*p ≤ 0.05; **p ≤ 0.01.

### Optimizing trait values of lentil ideotypes under different rainfall environments

**Figure 3:**
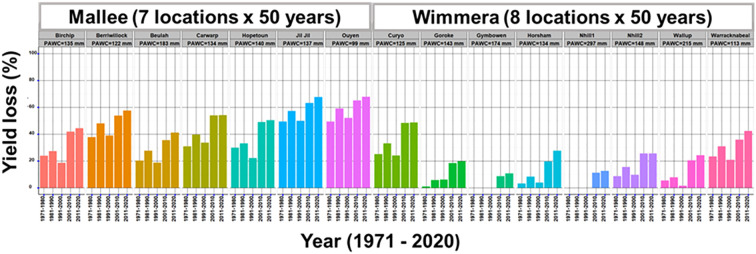
Predicted drought impact on lentils in Mallee and Wimmera environments of South-eastern Victoria.

In this study, the validated model was used to assess the impact of drought on lentil yield using 750 environments (15-locations and 50-years) between 1971 and 2020. Overall, lower yields were predicted in low rainfall (Mallee) than in medium rainfall (Wimmera) environments. Yield losses due to drought were shown to substantially increase over decades, particularly in the last two decades in all sites (**Figure 3**). The predicted average decadal yield losses were greater than 50% in medium rainfall (Wimmera) and 60% in low rainfall (Mallee) environments.

**Figure 4:**
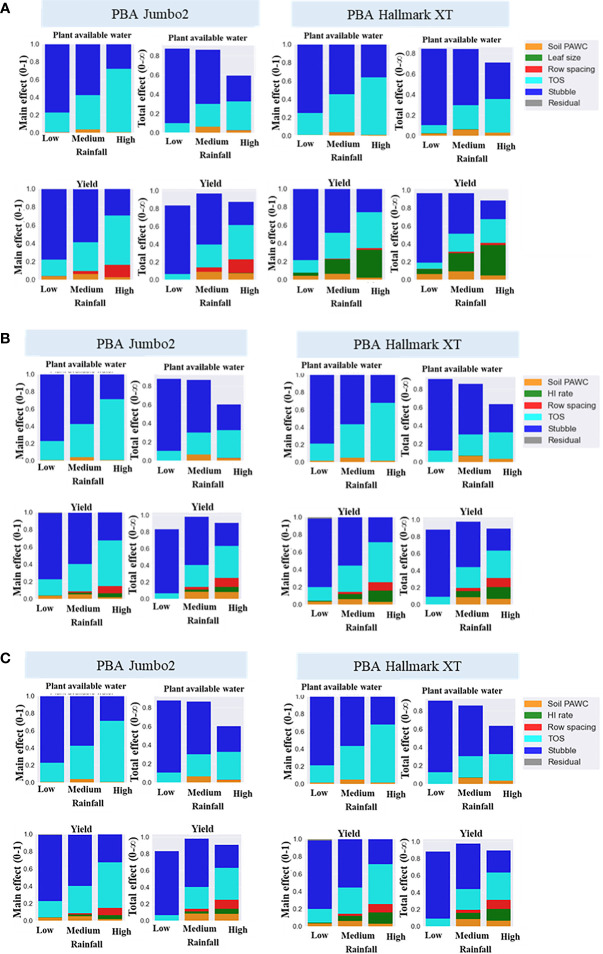
Main effect and total effect of stubble retention, time sowing (TOS), plant available water capacity (PAWC), leaf size **(A)** RUE **(B)**, HI rate **(C)** and row spacing explaining simulated plant available water seed yield variance in low, medium and high rainfall environments.

Further analysis was performed to quantify the impact of physiological traits and agronomic practices on plant available water and seed yield of lentils across different rainfall environments. **Figure 4** presents the main effect of physiological traits and agronomic practices explaining the plant available water and yield variance of lentils in low, medium and high rainfall environments. The total effect or the interaction effect, which explains the influence of one factor over the other factors is also presented in **Figure 4**. Stubble retention was the main contributor to the total variance, accounting over 70% of the variance in both plant available water and seed yield in water-limited environments (**Figure 4**). This effect generally declined with increased rainfall. In contrast, the proportion of the total variance explained by time of sowing and plant traits increased with increased rainfall. The contribution of the plant traits namely., leaf size, RUE and harvest index (HI) rate was greater in high than medium and low rainfall environments, but surprisingly this effect tended to decline with increased rainfall. Leaf size was found to be the highest contributor to seed yield followed by RUE and HI rate.

**Figure 5:**
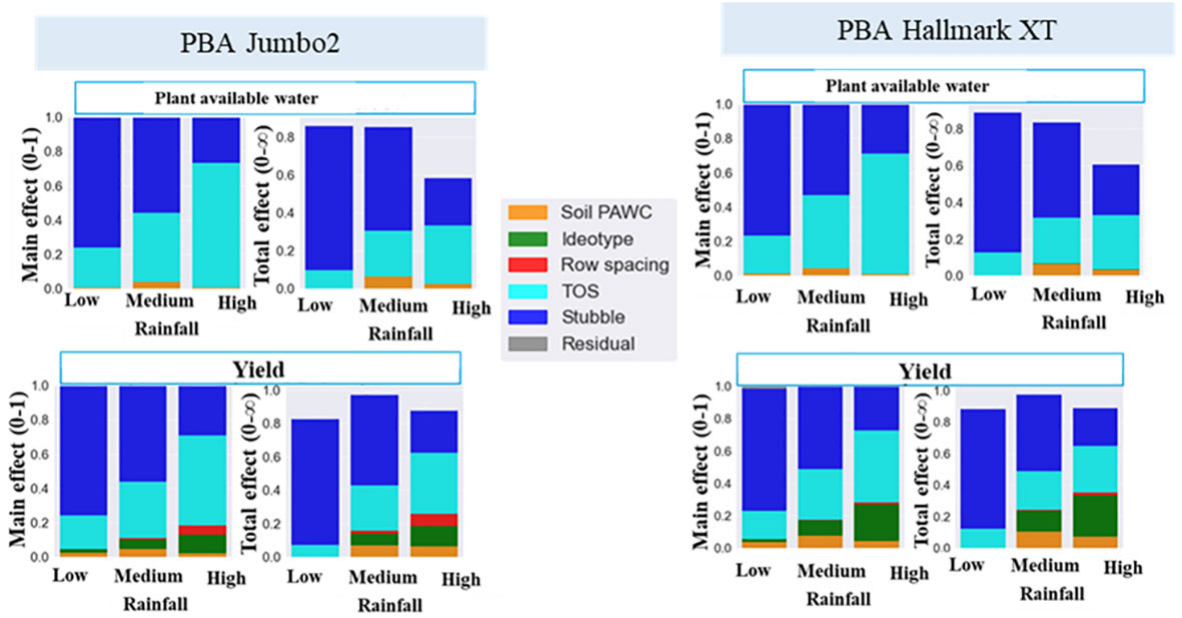
Drivers of plant available water and seed yield of lentils in low medium and high rainfall environments. PAWC, plant available water capacity; TOS, time of sowing. Ideotype was created by a ten percent increase (10%-40%) of individual trait (leaf size, RUE and HI rate) around baseline variety.

Main effect and total effect were further analyzed using ideotypes created from physiological traits (namely., leaf size, harvest index rate and radiation use efficiency) by a ten per cent increase around the baseline varieties, PBA Hallmark XT and PBA Jumbo2. The contribution of ideotype to plant available water and seed yield variance were greater than 20% and 15%, respectively (**Figure 5**). Soil type (PAWC) and row spacing were important in medium and high rainfall environments, respectively. In general, stubble retention was the main factor contributing to both water and yield variance in low rainfall environments and this effect decreased with increased rainfall. Time of sowing and ideotype were more important in high rainfall than in low and medium rainfall environments. High total effect values of ideotype and TOS in high rainfall zone shows higher influence of both factors on the remaining factors.

**Figure 6:**
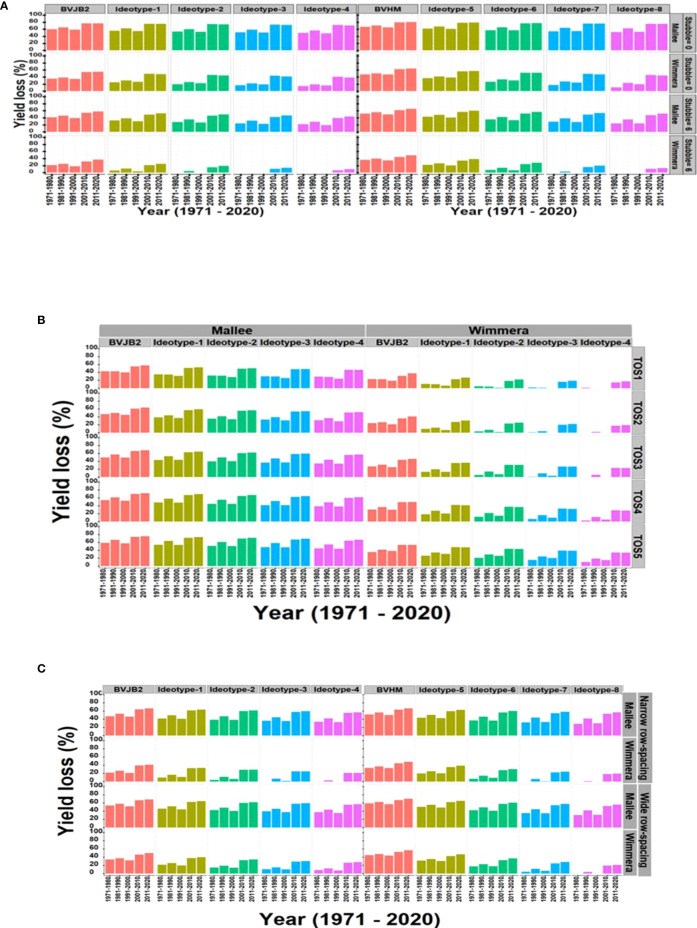
The synergies between ideotype and agronomic practices viz., stubble retention **(A)**, time of sowing **(B)** and row spacing **(C)** to reduce yield loss between 1971 and 2020 in low rainfall (Mallee) and medium rainfall (Wimmera) environments. Yield loss (%) was estimated in terms of average per decade. BVJB2, Baseline variety Jumbo2; BVHM, Baseline variety Hallmark; Ideotype was created by a ten percent increase (10%-40%) of individual trait (leaf size, RUE and HI rate) around baseline varieties; Ideotype 1 to 4 used genetic background of Jumbo2 and 5 to 8 used Hallmark; TOS, time of sowing. TOS1=April 28, TOS2=May 12, TOS3=May 26, TOS4=June 7, TOS5=June 23; Stubble treatments were 0 and 6 t/ha of initial surface residue of wheat.

**Figure 6A** presents what effect ideotypes and stubble retention had on maintenance of yield in dry seasons in Mallee and Wimmera environments. For example, the impact of drought was predicted to decline by up to 5% in Mallee and 25% in Wimmera by adopting improved physiological traits relative to baseline varieties. Wheat stubble of 6-t/ha reduced yield losses by up to 20% in low rainfall (Mallee) and 15% in medium rainfall (Wimmera) environments relative to the baselines. Combining both strategies reduced yield losses by 20% and 40% in low and medium rainfall environments, respectively.

Predicted yield losses were decreased by early sowing and growing improved ideotypes in both Mallee and Wimmera environments (**Figure 6B**). For example, yield losses were predicted to be 12% and 20% compared to late May and early June time of sowings. Likewise, combinations of reduced row spacing and physiological traits reduced yield loss in Wimmera. In general, the narrow row spacing slightly reduced yield loss compared to wide row spacing (**Figure 6C**).

## Discussion

The APSIM lentil module was used to identify traits that reduced the negative effects of drought as RUE, HI rate and leaf size (**Table 2** and **Supplementary Table 2**). Furthermore, interrogation of 50 years of climate data showed that the more recent two decades suffered from increased drought stress (**Figure 3**). These factors point to the requirement to breed for improved drought tolerance and outlines potential ideotypes to address this constraint.

### Genetic drivers of lentil yield

Our systematic approach of varying the RUE, HI rate and leaf size to explore eight potential ideotypes showed that these traits were not additive in terms of building an ideotype for high yield under drought. Nevertheless, we identified a combination that promises a yield advantage of around 10% against our current cultivars PBA Hallmark XT and PBA Jumbo2. Under drought conditions our ideotypes achieved 5 to 25% expected yield advantage in the absence of stubble residues and 20 to 40% yield advantage in their presence. This shows the importance of genetic screening under realistic production conditions (e.g., stubble retention in particular environments). Such screening is aided by the employment of biophysical models that incorporate both genetic and agronomic variables that focus on successful traits in combination, to reduce the impact of drought in the development of new cultivars for various environments. Traditionally, plant breeders select high yielding breeding lines for germplasm enhancement and variety development through long cycle recurrent selection approaches. This method is not only resource inefficient but also very expensive in development of varieties. Our coupled modelling approach is a powerful decision-making tool to aid breeders in defining traits of interest and varietal choice for targeted environments and production constraints as advocated by others for different crops (Hammer et al., 2006; Sharma et al., 2013; Rotter et al., 2015; Kaloki et al., 2019).

The current study presents a summary of cultivar specific coefficients derived from field experiments for commercially released varieties of lentil crop. To the best of our knowledge, this is the first study to measure and present more detailed variety specific harvest index rates, RUE, leaf size and phenology to be used in the APSIM-lentil cultivar module. We found clear differences among varieties evaluated for the plant traits and this is a baseline information to be used for further application in the APSIM-lentil model. Overall, the APSIM-lentil model simulated phenology, biomass, and seed yield with a good accuracy across our testing sites. This finding is consistent with previous results of modelling phenology and yield in lentil (Lake et al., 2021).

The proposed lentil ideotypes in two different physiological backgrounds reduced yield loss more in medium than in low rainfall environments, indicating the importance of physiological traits in maximizing yield in environments of higher rainfall. Stubble retention decreased yield loss more in low than medium rainfall environments relative to the baseline varieties. The synergies between both ideotype and stubble retention reduced yield loss by twofold in medium over low rainfall environments. Previous studies have also shown the adaptation of an ideotype and agronomic practice to drought in different crops (Hammer et al., 2006; Sharma et al., 2013; Rotter et al., 2015; Kaloki et al., 2019; Senapati and Semenov, 2019).

The combination of ideotypes and early time of sowing also reduced yield losses to drought in both low and medium rainfall environments. In other studies, delays of sowing by a week in this region had a yield penalty of 5-12% in canola crop (Farré et al., 2002; Kirkegaard et al., 2016) while adoption of early sowing strategy in Australia in general was estimated to contribute 7.1 Mt of wheat yield to the national production each year (Hunt et al., 2019). Overall, early sowing can be recommended to overcome the effects of terminal drought in several crops in this region and similar environments of Australia (Flohr et al., 2017; Flohr et al., 2018; Collins and Chenu, 2021).

Our result found early sowing (i.e., late April to first week of May) in lentils as the optimum window for most sites and growing seasons of low and medium rainfall environments of South-eastern Australia. Similar to our result, 25 April to 9 May of optimum sowing period of wheat was recommended for Wimmera and Mallee regions (Flohr et al, 2017). Based on the current climatic analysis, the combinations of early sowing with adaptive traits of ideotype more likely would allow lentil crops to provide an optimum flowering window for better yield performance under current and future drought conditions. In addition, adjustment of row spacing together with improved physiological traits reduced yield loss in medium rainfall environment in the present study. Overall, the narrow row spacing would cope with drought effects better than wide row spacing, mainly in drier environments. However, more comprehensive climatic analyses are needed to access the likely endurability of such ideotypes as our climate continues to change.

In general, lentil yield in low rainfall environments were largely improved due to improvements in agronomic practices, unlike findings in high rainfall environments. Improvement in physiological traits of leaf size, RUE and HI rate are regarded as the more promising traits related to yield improvement of lentils in high rainfall environments. Agronomic practices are the most adaptive strategy to reduce risks of drought in water limited environments. Moreover, the suitable match between agronomic practices and physiological traits are a useful adaptation approach to mitigate the impact of drought in lentils production and the current framework can be applied in other pulse crops for yield improvement.

Moreover, we have shown that increasing trait values in an ideotype can improve yield potential. Evidently, physiological trait based ideotype breeding can support selection for a conventional breeding method by first designing efficient combinations of plant traits that improve yield potential of lentils based on our current study; however, there is risk that some traits may be physiological difficult to achieve (e.g., a 40% increase in RUE). Clearly, some optimization of trait combination might be needed where all possible combinations including down-regulated traits are considered. This can potentially be supported by high throughput phenotyping and machine learning algorithms to make the method more efficient to achieve productivity advancement.

Ideotype breeding that combines phenological traits and agronomic practices is another potential area to optimize yield of lentils through minimizing the negative effects of production constraints. Crop growth model aided ideotype breeding is a useful approach to explore the optimal growing window of lentil by matching crop phenology with risk free periods of frost, heat, water and combinations of these stresses through varying the phenological traits of crop varieties in a given environment (Farré et al., 2002; Herndl et al., 2007; Chenu et al., 2011; Chenu et al., 2013; Hammer et al., 2014; Bell et al., 2015; Lilley et al., 2015; Chenu et al., 2017; Chauhan and Ryan 2020; Lake et al., 2021). For example, Herndl et al. (2007) presented a scenario analysis on the effect of sequencing phenological traits on the development and yield of wheat by avoiding the water stress period of the critical wheat growth stage in specific environments. Mousavi‐Derazmahalleh et al. (2019) also identified phenological traits for future climate change through an ideotype breeding approach and Rotter et al. (2015) developed early maturing ideotype by combining a set of phenological traits in wheat.

Understanding phenological traits is the first and the most basic step for ideotype design and development, where drought and other factors affect lentil productivity. However, characterization of lentil for phenological traits still requires a significant effort. In this study, we characterized the phenological traits of six varieties of lentils and these traits can be used for ideotype design and testing to maximize yield of lentils under abiotic production constraints. However, crop growth model based ideotype breeding lacks the capability for predicting the effects of biotic factors such as disease, insect pests as well as quality traits for ideotype breeding (Keating et al., 2003; Holzworth et al., 2014).

### Agronomic drivers of lentil yield

Recent progress in process-based crop simulation modelling opens the way for better understanding of the drivers of crop productivity and the suitable match of physiological traits and agronomic practices to optimize yield (Webber et al., 2018; Ojeda et al., 2021; Ojeda et al., 2022). In the present study, the drivers of plant available water and seed yield of lentils were assessed using sensitivity analysis. Our study found stubble retention as a main factor explaining the largest proportion of the total variance of plant available water and seed yield of lentils in water-limited environments The influence of ideotype and time of sowing was very higher in high rainfall environment (**Figures 4**, **5**), indicating that these factors are highly interactive based on previous studies (Webber et al., 2018; Ojeda et al., 2021; Ojeda et al., 2022). However, ideotype and time of sowing had less frequency of cross over interactions than stubble retention in the current study.

The advantage of stubble retention in dry environment where soil water storage can be increased (e.g. in clay soils) is related to improved soil water infiltration, and reduced water loss to evaporation (O’Leary and Connor, 1997; Monzon et al., 2006; Roper et al., 2013; Page et al., 2019).

Conservation of water with stubble retention is not as critical in higher rainfall environments and a decline in the contribution of stubble retention on plant available water and yield is likely. Unlike low rainfall environments, time of sowing and physiological traits (leaf size, RUE, HI rate) were found to be important factors in non-droughted environments, while this effect tended to decline with decreased rainfall. In addition, medium to late maturing varieties could also be beneficial to increase crop productivity in high yielding environments (O’Leary and Connor, 1985; Hunt, 2017; Rahimi-Moghaddam et al., 2021).

In drier environments, large leaf size and fast leaf development were good traits for early vigor and better weed control during early crop growth. In addition, leaves are the primary part of a plant for photosynthesis, where plants maximize the use of the intercepted photosynthetic active radiation (PAR) and convert water and carbon dioxide into biomass and yield in high resource input environments. A large leaf size has been related to high photosynthetic capacity of individual leaf (Long et al., 2006; Wu et al., 2018) in high rainfall environments.

### Cultivar specific parameterization

The current study computed node appearance, which varied with varieties from 57 to 61°Cd. However, in the APSIM version 7.10, the default value for the node appearance rate of lentil has been set at 61°Cd regardless of varietal differences. Based on our results, the node appearance on the main stem tended to have a faster rate for stay green late maturing varieties, such as Nipper, PBA ACE, and PBA Hallmark XT. Leong and Ong (1983); Robertson et al. (2002) and Sato (1971) reported a node appearance rate of 47°Cd for chickpea, 101°Cd for mungbean, 56°Cd for Peanut and 51°Cd for Lucerne. Robertson et al. (2002) also showed a strong and positive association between the number of nodes and total leaves per plants in chickpea (R2 = 0.84) and mungbean (R2 = 0.82). Early node development at a faster rate is important for early canopy development and is expected to improve radiation interception and seed yield (Bonnett, 1998).

The phenological development in the APSIM-lentil model is defined by eleven growth stages. In this study we have characterized the key phenological phases of lentil as required in the APSIM framework. The lag phase, a slow growth phase of shoot elongation between sowing and germination is driven by cultivar specific fixed thermal time (Wu et al., 2018; Roberstson and Lilley 2016). A cumulative thermal time of 60.80°Cd of lentil shoot lag at sowing depth of 50 mm was calculated from measured field data. A linear growth phase of lentil shoot elongation between lag and seedling emergence showed a faster growth rate (2.42°Cd/day) in the current study. In the model this rate is linearly related to temperature and sowing depth (Robertson et al., 2002; Keating et al., 2003; Holzworth et al., 2014; Roberstson and Lilley, 2016).

The current study also used data from field experiments and described phenological stages of lentil as defined in APSIM. The duration between emergence and end of juvenile phase is driven by thermal time. We calculated growth phases from emergence to end of juvenal phase and from end of juvenile phase to floral initiation in terms of cumulated thermal time. Measured duration between stages varied with cultivar, where emergence to end of juvenile ranged between 559 °Cd and 681 °Cd, end of juvenile to floral initiation ranged between 116 °Cd and 277 °Cd and start pod fill to end of pod fill ranged between 545 °Cd and 679 °Cd. Unlike our results, a recent study by Lake et al. (2021) adjusted the default values of lentil phenology based on a sensitivity analysis and used 700 °Cd for the end of juvenile to floral initiation, 446 °Cd for floral initiation to flowering and 690 °Cd for the start to end of pod fill in lentil. The discrepancies between these studies could be due to the insufficient parameterization (Roberstson and Lilley, 2016) in the Lake et al. (2021) study.

To the best of our knowledge, this is the first study to measure and present variety specific harvest index rates to be used in the APSIM-lentil model module. We found clear differences among varieties evaluated for harvest index rate. Studies have shown improvement in seed yield due to increase in HI (Koester et al., 2014; Suhre et al., 2014; Flohr et al., 2018) and this can be used as an indicator of reproductive efficiency. The APSIM model uses the simulated daily biomass for partitioning to seed yield, pod wall, stem, and leaf developments. The field measured harvest index rate of six varieties of lentils ranged from 0.0090 to 0.0139 per day. Moot et al. (1996) reported harvest index rate of wheat between 0.0099 and 0.0103 per day. Further study by Fletcher and Jamieson (2009) found the values of harvest index rate between 0.0058 and 0.0164 per day in wheat. Using crop simulation modelling, a yield increase of wheat ideotype was reported because of increased harvest index under drought condition (Semenov et al., 2014). Xiao et al. (2020) and Sexton et al. (2017) also found significant relationships between yield and RUE in maize and sugarcane.

Overall, modern varieties had higher genotypic coefficients of physiological traits. From this result, it appears that the modern-day varieties yield gain could be driven by improvement in physiological traits.

## Conclusion

APSIM-lentil model was parameterized for the first time in detail to improve model prediction. The stepwise process of parametrization employed provided robust cultivar specific coefficients that are suitable for wider ideotype investigations with the APSIM-lentil model. The stepwise process required initial measured soil water at our experimental sites utilizing nearby APSoil parameters. Overall, the APSIM-lentil model performed well in predicting phenology, seed yield and biomass and the model should be applicable to other soil types of South-eastern Australia and possibly other similar environments. A better understanding of ideotype and agronomic practices synergies using crop modelling helped to identify high yield potential of lentil crops in specific environments. Agronomic options (sowing date, stubble retention and row spacing) together with improved physiological traits can be used to reduce the impact of drought stress in lentils. In a low rainfall environment, stubble retention and time of sowing are more important than physiological traits/ideotypes. The ideotype concept in lentil breeding should significantly support breeders to define traits of interest and varietal choice for targeted environments and production constraints. Unlike in a low rainfall environment, selection for physiological traits is important for yield improvement in lentil crops grown in medium and high rainfall environments. The present study identified ideotype designing coupling with agronomic practices adjustment as a very important adaptation strategy to reduce the impact of drought.

## Data availability statement

The original contributions presented in the study are included in the article/**Supplementary Material**. Further inquiries can be directed to the corresponding author. The APSIM software and documentation is publicly available.

## Author contributions

Conceptualization and methodology, GR, GO’L, and AT. Funding acquisition and supervision, GR. Data collection, analysis, modelling and writing of the first draft, AT. Revisions, GO’L, GR, and RA. Assisted in crop modelling, GO’L and TT. Assisted in data collection, SR and VS-P. Edition and comments, all authors. All authors contributed to the article and approved the submitted version.

## Funding

The study was supported by Agriculture Victoria and the Grain Research and Development Corporation.

## Acknowledgments

We thank the Agriculture Victoria (AV) and the Grain Research and Development Corporation (GRDC), Australia for supporting this project (Project VGIP1B- IDGRDC 8049295), Physiological and agronomic approaches to achieve transformational productivity improvements in pulses in southern Australia and contribution from associated project VGIP2B Intercropping to exploit rainfall for profit. We also thank the Molecular Plant Breeding team of Horsham Grain Innovation Park for their technical support, particularly to the VGIP1B team, Tamika Mentha, Jignesh Vakani and Sunita Bastakoti Pandey.

## Conflict of interest

The authors declare that the research was conducted in the absence of any commercial or financial relationships that could be construed as a potential conflict of interest.

## Publisher’s note

All claims expressed in this article are solely those of the authors and do not necessarily represent those of their affiliated organizations, or those of the publisher, the editors and the reviewers. Any product that may be evaluated in this article, or claim that may be made by its manufacturer, is not guaranteed or endorsed by the publisher.
