# Supplementary material for: Identification of agro-physiological traits of lentil that reduce risks of drought
Source: Front Plant Sci. 2022 Oct 24;13:1019491. doi: 10.3389/fpls.2022.1019491 (PMC9637959; doi:10.3389/fpls.2022.1019491)
Supplement: Supplementary file 1 [file DataSheet_1.docx]

Supplementary Material

**Supplementary Table 1:** Soil specific model parameters

| **Soil Depth** | **BD** | **Air dry** | **LL15** | **DUL** | **SAT** | **NH_4_** | **NO_3_** | **P** | **pH** | **EC** | **TC** | **Total N** | **ESP** | **Exchangeable bases** | | | | **S** | **Cu** | **Fe** | **Mn** | **Zn** | **Al** | **B** | **Cl** | **Soil particle size** | | |
| --- | --- | --- | --- | --- | --- | --- | --- | --- | --- | --- | --- | --- | --- | --- | --- | --- | --- | --- | --- | --- | --- | --- | --- | --- | --- | --- | --- | --- |
|  |  |  |  |  |  |  |  |  |  |  |  |  |  | **Ca** | **Mg** | **Na** | **K** |  |  |  |  |  |  |  |  | **(%)** | | |
| **cm** | **gcm^-3^** | **--------------mm mm^-1^---------------** | | | | **mg/kg** | **mg/kg** | **mg/kg** | **CaCl2)** | **dS/m** | **%** | **%** | **%** | **meq/100g** | **meq/100g** | **meq/100g** | **meq/100g** | **mg/kg** | **mg/kg** | **mg/kg** | **mg/kg** | **mg/kg** | **mg/kg** | **mg/kg** | **mg/kg** | **Clay** | **Sand** | **Silt** |
| **Hopetoun, Low-Rainfall Zone** | | | | | | | | | | | | | | | | | | | | | | | | | | | | |
| 0-10 | 1.350 | 0.065 | 0.068 | 0.236 | 0.421 | 6.67 | 18.00 | 57.00 | 6.27 | 0.17 | 1.02 | 0.10 | 1.06 | 6.61 | 1.76 | 0.10 | 0.93 | 77.80 | 0.72 | 16.77 | 10.91 | 2.40 | 0.35 | 1.87 | 7.03 | 13.12 | 78.98 | 7.90 |
| 10-30 | 1.290 | 0.157 | 0.157 | 0.342 | 0.443 | 1.33 | 6.00 | 10.33 | 7.83 | 0.22 | 0.36 | 0.04 | 1.53 | 14.66 | 6.00 | 0.33 | 0.64 | 43.13 | 0.80 | 13.37 | 1.78 | 0.54 | 0.21 | 3.14 | 5.70 | 22.87 | 61.16 | 15.97 |
| 30-50 | 1.290 | 0.191 | 0.191 | 0.404 | 0.443 | 1.00 | 8.33 | 5.00 | 8.40 | 0.53 | 0.21 | 0.02 | 10.16 | 15.24 | 9.89 | 2.94 | 0.86 | 44.00 | 1.12 | 14.53 | 0.89 | 0.41 | 0.29 | 18.92 | 16.45 | 37.46 | 45.05 | 17.49 |
| 50-70 | 1.370 | 0.187 | 0.187 | 0.367 | 0.413 | 1.00 | 7.67 | 1.67 | 8.60 | 0.46 | 0.12 | 0.02 | 7.31 | 12.81 | 8.71 | 1.75 | 0.66 | 74.23 | 0.88 | 8.67 | 0.59 | 0.72 | 0.24 | 17.06 | 91.97 | 31.93 | 56.67 | 11.40 |
| 70-90 | 1.370 | 0.197 | 0.197 | 0.382 | 0.398 | 1.00 | 6.33 | 1.67 | 8.73 | 0.57 | 0.09 | 0.01 | 10.24 | 10.92 | 6.75 | 2.08 | 0.57 | 119.47 | 0.82 | 8.97 | 0.75 | 0.44 | 0.24 | 19.91 | 142.23 | 32.91 | 54.64 | 12.45 |
| 90-110 | 1.410 | 0.191 | 0.198 | 0.396 | 0.394 | 1.00 | 5.33 | 1.67 | 8.57 | 0.58 | 0.09 | 0.01 | 15.86 | 8.43 | 6.05 | 2.84 | 0.59 | 136.00 | 0.81 | 6.87 | 0.44 | 0.29 | 0.24 | 19.74 | 169.73 | 34.47 | 53.71 | 11.82 |
| 110-130 | 1.420 | 0.191 | 0.191 | 0.393 | 0.393 | 1.00 | 5.33 | 1.67 | 7.70 | 0.47 | 0.10 | 0.01 | 16.74 | 6.00 | 4.29 | 2.15 | 0.40 | 123.20 | 0.84 | 9.33 | 0.43 | 0.41 | 0.24 | 18.82 | 201.60 | 31.93 | 55.10 | 12.97 |
| **Horsham, Mid-Rainfall Zone** | | | | | | | | | | | | | | | | | | | | | | | | | | | | |
| 0-10 | 1.150 | 0.115 | 0.258 | 0.445 | 0.496 | 1.00 | 20.00 | 30.00 | 7.43 | 0.23 | 1.28 | 0.08 | 0.82 | 25.51 | 5.95 | 0.28 | 1.81 | 4.93 | 0.93 | 19.93 | 4.17 | 1.05 | 0.25 | 3.03 | 5.47 | 23.28 | 39.93 | 36.81 |
| 10-30 | 1.136 | 0.196 | 0.311 | 0.465 | 0.501 | 1.33 | 20.00 | 2.67 | 8.03 | 0.38 | 1.09 | 0.05 | 5.70 | 25.27 | 8.64 | 2.13 | 1.36 | 5.60 | 1.30 | 21.77 | 1.43 | 0.26 | 0.23 | 5.25 | 66.53 | 50.40 | 31.95 | 17.65 |
| 30-50 | 1.119 | 0.267 | 0.390 | 0.397 | 0.508 | 1.67 | 15.33 | 2.33 | 7.97 | 0.30 | 0.72 | 0.05 | 9.53 | 21.32 | 9.45 | 3.38 | 1.30 | 3.90 | 1.54 | 23.63 | 1.76 | 0.22 | 0.22 | 5.98 | 24.83 | 55.53 | 27.67 | 16.80 |
| 50-70 | 1.157 | 0.260 | 0.319 | 0.405 | 0.493 | 1.67 | 15.33 | 2.00 | 8.20 | 0.42 | 0.91 | 0.04 | 12.81 | 19.01 | 10.64 | 4.54 | 1.26 | 9.53 | 1.50 | 24.13 | 1.55 | 0.24 | 0.23 | 9.83 | 75.70 | 56.54 | 27.99 | 15.47 |
| 70-90 | 1.164 | 0.256 | 0.305 | 0.405 | 0.491 | 1.67 | 18.67 | 3.33 | 8.00 | 0.44 | 0.88 | 0.04 | 12.08 | 17.31 | 9.69 | 3.88 | 1.25 | 38.53 | 1.39 | 21.33 | 2.09 | 0.38 | 0.23 | 12.56 | 112.53 | 51.38 | 32.06 | 16.56 |
| 90-110 | 1.128 | 0.244 | 0.307 | 0.443 | 0.504 | 2.00 | 11.00 | 3.00 | 8.13 | 0.62 | 0.90 | 0.02 | 18.14 | 14.14 | 11.07 | 5.87 | 1.28 | 78.63 | 1.38 | 21.60 | 1.63 | 0.33 | 0.21 | 13.42 | 286.07 | 53.05 | 28.13 | 18.82 |
| 110-130 | 1.137 | 0.257 | 0.298 | 0.444 | 0.501 | 1.33 | 7.67 | 2.33 | 8.23 | 0.57 | 1.75 | 0.02 | 12.69 | 15.38 | 11.61 | 4.08 | 1.09 | 23.63 | 1.24 | 15.87 | 1.14 | 0.26 | 0.24 | 14.63 | 268.23 | 50.73 | 34.77 | 14.49 |

**Supplementary Table 2**: Field measured cultivar specific model parameters of six lentil cultivars.

| **Parameter** | **Unit** | **Range** | | |
| --- | --- | --- | --- | --- |
|  |  | **Cultivars** | **Ideotypes developed from** | |
|  |  |  | **Jumbo2** | **Hallmark XT** |
| TT from emergence to end of juvenile phase | ^o^Cd | 559 - 627 | 559 - 627 | |
| estimated days from emergence to floral init. | days | 96 - 99 | 96 - 99 | |
| TT from end juvenile to floral initiation | ^o^Cd | 116 - 240 | 116 - 240 | |
| TT from initiation to flowering | ^o^Cd | 70 - 218 | 70 - 218 | |
| TT from flowering to start grain fill | ^o^Cd | 245 - 391 | 245 - 391 | |
| TT from start grain fill to end grain fill | ^o^Cd | 617 - 690 | 617 - 690 | |
| TT from end grain fill to maturity | ^o^Cd | 60 | 60 | |
| TT from maturity to harvest ripe | ^o^Cd | 1 | 1 | |
| RUE | g/MJ | 0.6700-0.9000 | 0.99 – 1.26 | 0.88 – 1.12 |
| Node appearance rate | #/^o^Cd | 0.0165-0.0174 | 0.0154-0.0121 | 0.0159-0.0125 |
| Harvest index rate | (g/g)/d | 0.0028 - 0.0139 | 0.014-0.017 | 0.010-0.013 |
| Leaf size at juvenile | cm^2^ | 180 - 206 | 227-288 | 209-266 |
| Leaf size at floral initiation | cm^2^ | 525 - 693 | 749-953 | 578-735 |
| shoot lag = 60.80 ^o^Cd | | | | |
| shoot rate = 2.42 ^o^Cd/mm per depth | | | | |

TT=thermal time; shaded table is field measured parameters. RUE parameter for APSIM-lentil model based on intercepted total solar radiation


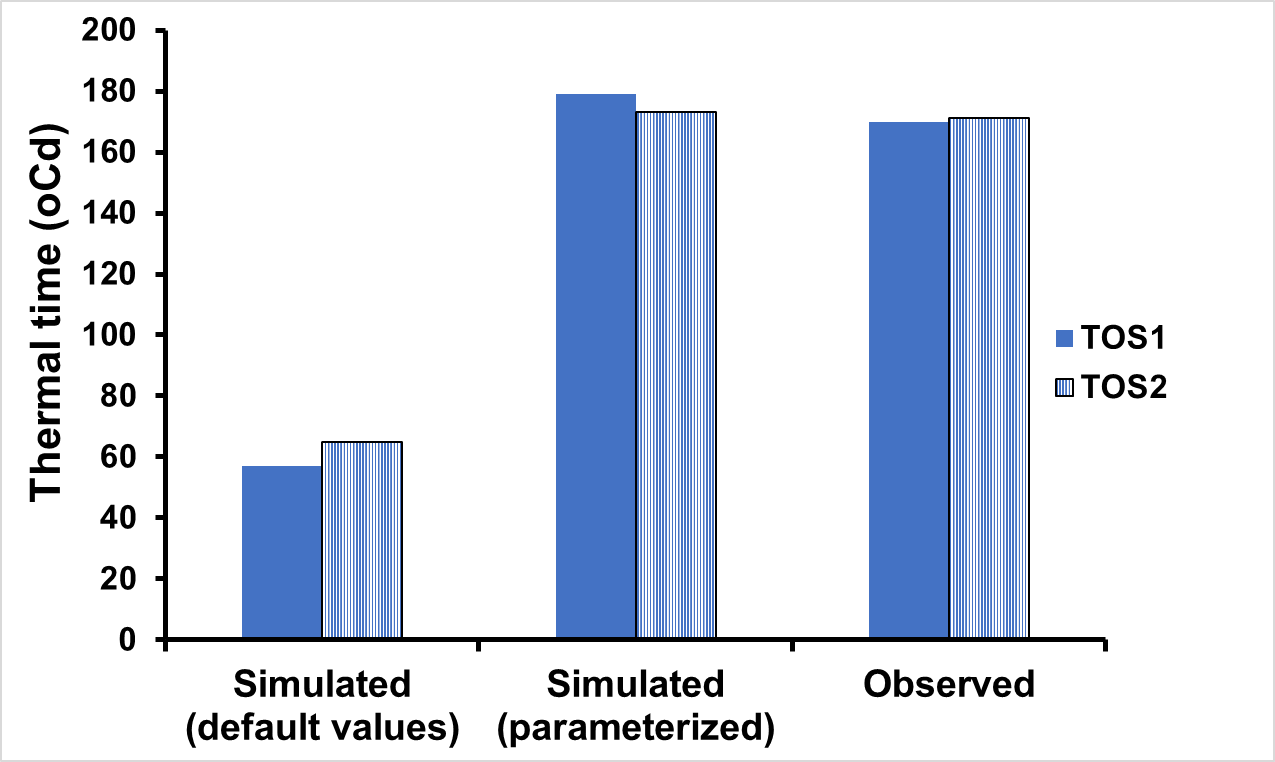


**Supplementary Figure 1**: Simulated and measured days to emergence (^o^Cd)


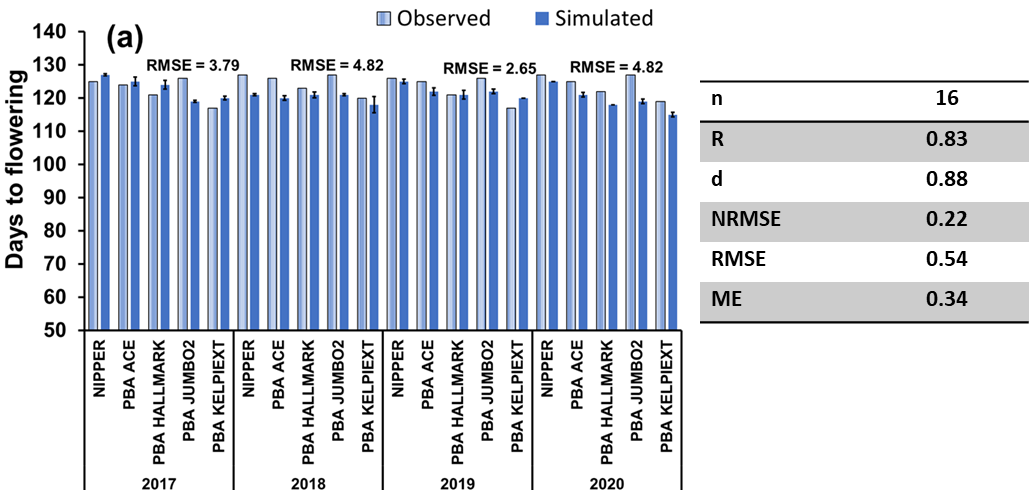


***
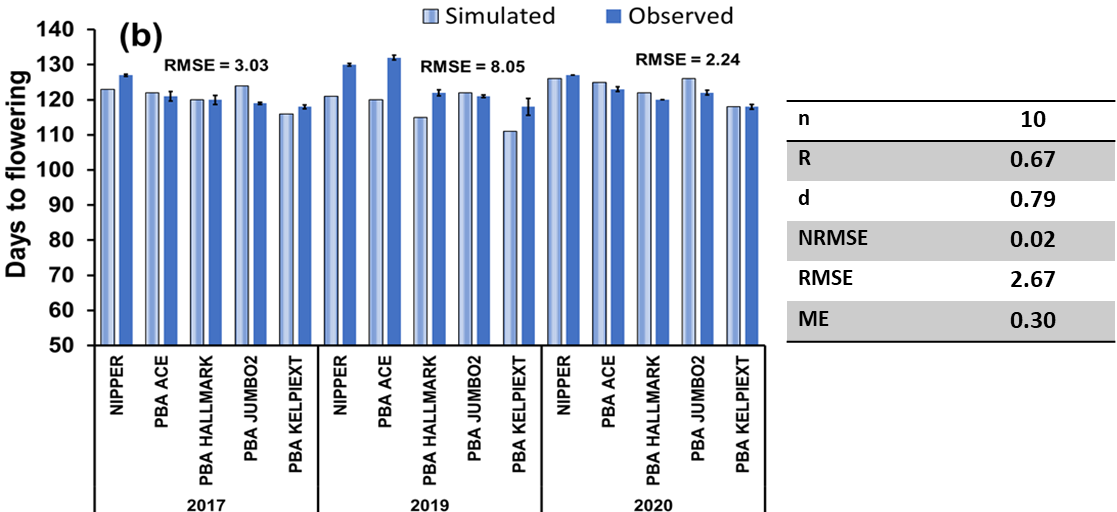
***

***
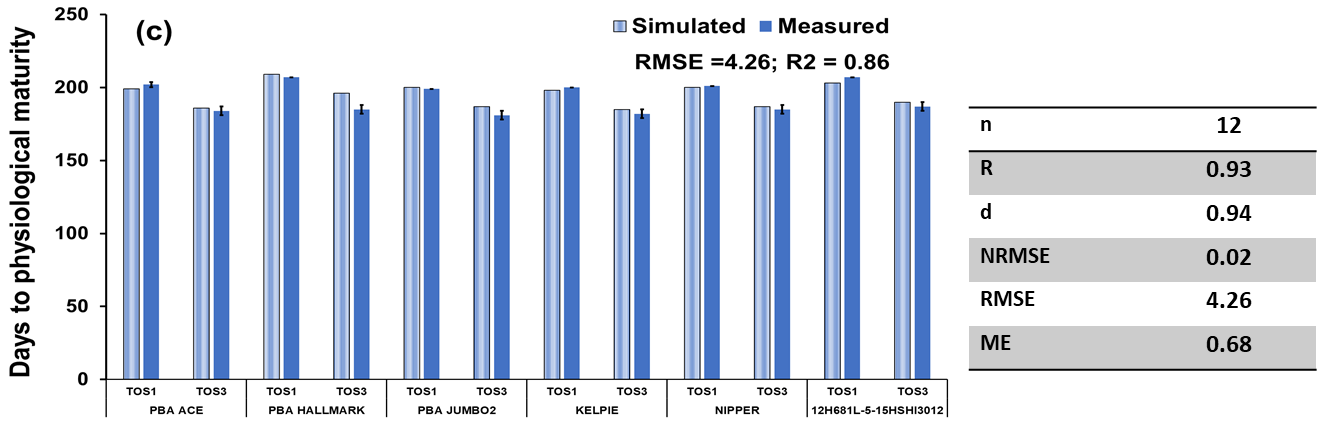
***

**Supplementary Figure 2:** Simulated and measured days to flowering at Horsham (a) and Beulah (b) and physiological maturity at Horsham (c) of six lentils genotypes


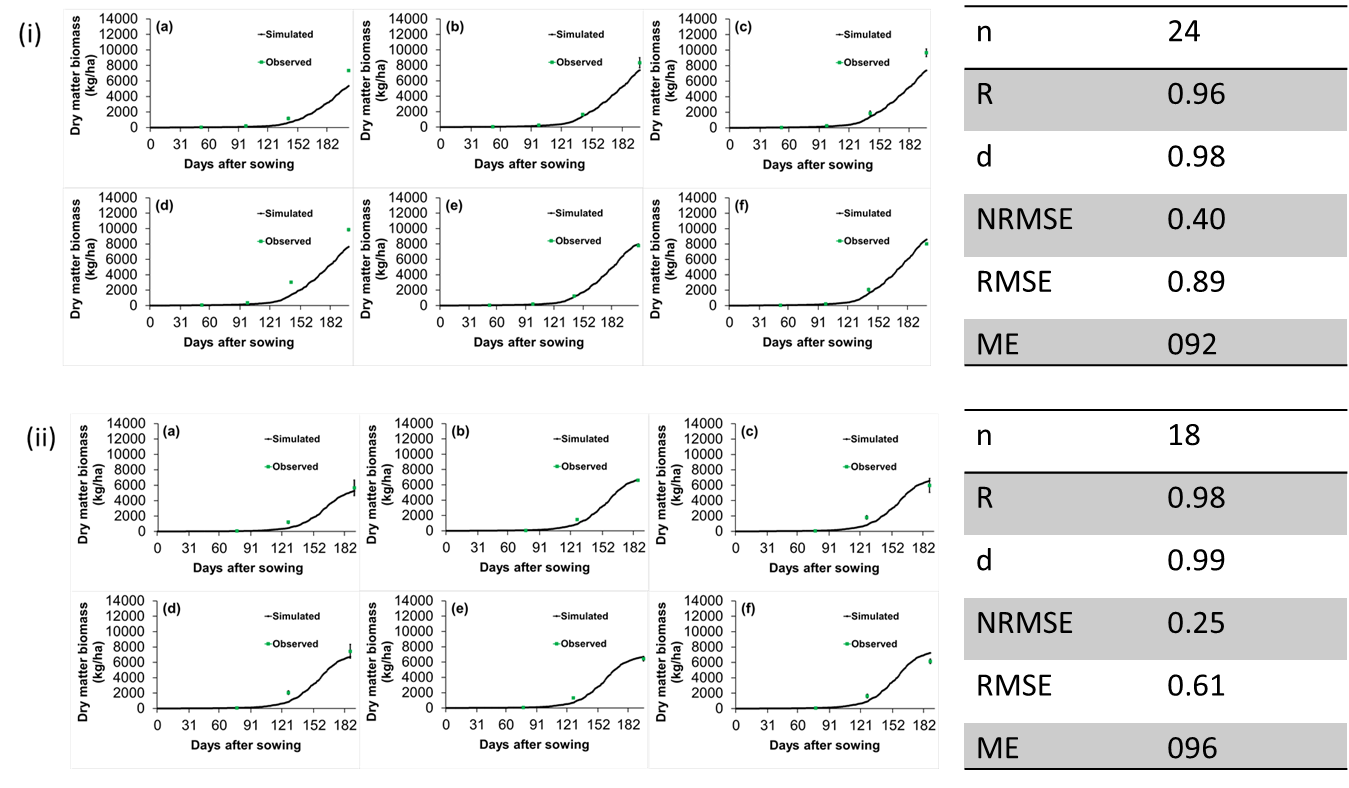


**Supplementary Figure 3:** The relationship between simulated and observed aboveground biomass of six lentil varieties (i) =TOS1; (ii)=TOS3, (a)=12H681L-5-15HSHI3012, (b)=PBA KELPIE, (c)=NIPPER, (d)=PBA ACE, (e)=PBA HALLMARK, (f)=PBA JUMBO2
